# Virtual screening of novel compounds as potential ER-alpha inhibitors

**DOI:** 10.6026/97320630015321

**Published:** 2019-04-30

**Authors:** Jakkanaboina TilakVijay, Kandimalla Vivek Babu, Addepally Uma

**Affiliations:** 1 Centre for Biotechnology, Institute of Science and Technology, Jawaharlal Nehru Technological University, Hyderabad, Telungana, India

**Keywords:** molecular docking, virtual screening, ERa, estrogen, bipyrazoles, drug Bank

## Abstract

Majority of breast cancers diagnosed today are estrogen receptor (ER)-positive, however, progesterone receptor-positive (PR-positive) is also responsible for breast cancer. Tumors that are ER/PR-positive are much more likely to respond to hormone therapy than tumors that are ER/PR-negative. Nearly 105 ERa inhibitors from literature when docked resulted in 31 compounds (pyrazolo[1,5-a]pyrimidine analogs and chromen-2-one derivatives) with better binding affinities. The maximum score obtained was -175.282 kcal/mol for compound, [2-(4- Fluoro-phenylamino)-pyridin-3-yl]-{4-[2-phenyl-7- (3, 4, 5-trimethoxy-phenyl)-pyrazolo[1,5-a]pyrimidine-5-carbonyl]-piperazin-1-yl}-methanone. The major H-bond interactions are observed with Thr347. In pursuit to identify novel ERa inhibitory ligands, virtual screening was carried out by docking pyrazole, bipyrazole, thiazole, thiadiazole etc scaffold analogs from literature.34 bipyrazoles from literature revealed Compound 2, ethyl 5-amino-1-(5-amino-3-anilino-4-ethoxycarbonyl-pyrazol-1-yl)-3-anilino-pyrazole-4-carboxylate, with -175.9 kcal/mol binding affinity with the receptor, where a favourable H-bond was formed with Thr347.On the other hand, screening 2035 FDA approved drugs from Drug Bank database resulted in 11 drugs which showed better binding affinities than ERa bound tamoxifen. Consensus scoring using 5 scoring schemes such as Mol Dock score, mcule, SwissDock, Pose&Rank and DSX respectively resulted in better rank-sumsfor Lomitapide, Itraconazole, Cobicistat, Azilsartanmedoxomil, and Zafirlukast.

## Background

Majority of breast cancers diagnosed today are estrogen receptor (ER)-positive, where, estrogen binds to estrogen receptors on the surface of the cell 1. According to the American Cancer Society, about 2 out of every 3 cases of breast cancer is hormone receptorpositive. However, in certain cases, progesterone receptor-positive (PR-positive) is also responsible for breast cancer 2. Tumors that are ER/PR-positive are much more likely to respond to hormone therapy than tumors that are ER/PR-negative. ERa-positive breast cancer is more resistant to chemotherapy than ERa-negative cancer 3. Estrogen- receptor status and outcomes of modern chemotherapy for patients with node-positive breast cancer is known. ERa plays an important role in determining the sensitivity of breast cancer cells to chemotherapeutic agents in vitro 4. Down regulation of Aurora-A overrides estrogen-mediated growth and chemo resistance in breast cancer cells. Patients with ER-a-positive tumors have a slightly better survival rate than patients with ER-a- negative. However, both the ER and PR respond to the drug tamoxifen, designed to interfere the function of ER-a 5. Tamoxifen decreases the incidence of invasive and non-invasive breast cancer. In spite of the tamoxifen administered side effects, its use as a breast cancer preventive agent is appropriate in many women at increased risk for the disease 6. ER-a is thought to function as a ligand-activated transcription factor. Extracellular signals can also stimulate ER-a-mediated transcription in the absence of estrogen. Stimulated ER-a can influence gene expression by associating with other transcription factors without binding directly to DNA Estrogen receptor alpha rapidly activates the IGF-1 receptor pathway 7-8. Specific binding sites for estrogen at the outer surfaces of isolated endometrial cells are known. Estrogens stimulate growth of many breast cancer cells. Reducing estrogen levels or blocking often leads to a clinical response in patients with receptor-positive disease. In premenopausal women, estrogen production is high and in postmenopausal women relatively small amounts of estrogens are produced. These low levels of estrogens can be inhibited either by blocking the estrogen receptor, or by inhibiting the peripheral conversion of androgens to estrogens 9. The most widely accepted pharmacologic endocrine therapies for breast cancer are treatment with anti estrogens 10. Tamoxifen has been shown to be effective in both premenopausal women as well as in postmenopausal women 11. Tamoxifen is the most widely used and extensively studied anti estrogen and its role in the management of patients with breast cancer is well established 12. However, extensive evaluation of tamoxifen treatment revealed significant side effects such as endometrial cancer, blood clots and the development of acquired resistance. Hence, there is a pressing need for the improvement and/or development of new antiestrogens for the prevention and treatment of breast cancer.

## Methodology

A search for Estrogen Receptor alpha (ER-a) structure in Protein Data Bank (PDB) [www.rcsb.org/pdb] revealed several hits with bound ligands and drugs. In general, the selection of the receptor is based on highest possible resolution, no mutations or modified residues and the presence of bound ligand or drug 13 in particular. The resolution ensures that 3D structures utilized for docking were of a good quality and on the other hand, the structure should be devoid of any mutations, this is because mutations might have profound effects on the final confirmation of a protein 14 15. Moreover, a co-crystallized bound ligand represents better geometric orientation within the active site space of the protein. Therefore, the 3D structure of ERa bound with an antagonist, i.e. 4- hydroxytamoxifen (PDB ID: 3ERT), was selected as the preferred docking target protein.

### Molecular Docking Analysis:

Molecular docking is a study of non-bonded, non-covalent interactions between a receptor or active site region of a protein and a drug or chemical molecule forming an intermolecular complex 16. Docking is carried out to dock various conformations of small molecules to a receptor followed by evaluation of the molecules with respect to the geometrical orientation and complementarity in terms of shape and properties, such as electrostatics 17. The outcome of a docking routine includes affinity prediction (scoring) for the molecules investigated, yielding a relative rank ordering of the docked compounds with respect to affinity, reported as kcal/mol 18.

### Molegro Virtual Docker:

Molegro Virtual Docker is an integrated platform for predicting protein - ligand interactions 19. All default options including preparation of the molecules to determination of the potential binding sites of the target protein, and prediction of the binding modes of the ligands were employed.

### Ligand Drawing:

All ligands were drawn using ISIS-Draw (v. 2.3), which is a userfriendly drawing package that enables to draw chemical structures. ISIS/Draw is mainly a 2D drawing program with structure and reaction validation features and can calculate elementary properties such as formula and molecular weight 20 the 2-D structures are converted into 3-dimensional structures using ProDrug2 server 21.

### Datasets:

#### Set-1: ERa ligands from literature

Nearly 105 ligands reported as antagonists of ERa such as benzofurans 22, diphenyl amine analogs 23, sulfoximine-based acyclic triaryl olefins 24, isoxazole derivatives 25 thiazolidinone derivatives 26, tamoxifen mimics 27, pyrazolo[1,5-a]pyrimidine conjugates 28 chromen-2-one derivatives 29 etc. Many of those compounds are serving as anticancer agents 30 antifungal agents 31 and anti-inflammatory agents 32 etc. were selected for molecular docking analysis.

#### Set-2: ERa Non-tested ligands from literature

The method employed is to screen similar repertoire of inhibitors reported in various literature sources to identify new probable active compounds, which have not been tested for ERa inhibitory activity. Therefore search initiated for compounds containing pyrazole, bipyrazole, thiazole, thiadiazole etc scaffold analogs reported in Archives of organic chemistry journal www.arkat-usa.org. After preliminary docking investigations, bipyrazole classes of compounds were known to elicit inhibitory characteristics against ERa. Hence, a set of 34 bipyrazoles reported in literature www.arkat-usa.org was considered in the study 33-34. Set-3: Drugs from Drug-Bank Database The rationale to choose Drug Bank database is due to the larger collection and unique resource of drugs with detailed information on each drug and drug target. The latest release of Drug-Bank (version 5.0.10, released 2017-11-14) contains 10,555 drug entries including 1,745 approved small molecule drugs, 877 approved biotech (protein/peptide) drugs, 107 nutraceuticals and over 5,031 experimental drugs. Additionally, 4,775 non-redundant protein (i.e. drug target/enzyme/transporter/carrier) sequences are linked to these drug entries 35. In the present study, 2035 approved drugs were selected for analysis.

### Consensus scoring for enrichment of drugs:

In general, docking routines have the capability to correctly predict protein-ligand complex structures with rational accuracy which is determined based on the RMSD of docked ligand within active site space of the target protein. The ability to forecast the possible geometric binding mode of the docked ligand to distinguish exact poses from incorrect ones is dependent on various scoring functions. Therefore, as is evidenced that both docking analysis and scoring functions play vital importance in drug design procedures, it was reported that the weakness of docking programs is their built-in scoring functions. The main scoring functions include the knowledge-based 36, Physics-based 37, and empirical 38 scoring functions. Therefore, combining various scoring functions would certainly minimize the errors that appear in single scoring programs and thereby enhance the chance of recognizing true hits 39. Thus, it has been demonstrated that consensus scoring is generally more effective than single scoring for molecular docking 40 and represented an effective way in getting improved hit rates in various virtual database screening studies 41. In this study, about five scoring functions were employed to evaluate consensus scoring patterns, they are: MolDock score of Molegro, Swiss Dock, mcule docking paradigm, Pose & Rank scoring, DSX scoring schemes respectively. Classes were generated based on the dock scores followed by ranking the best conformations.

## Results and Discussion

The crystal structure of human estrogen receptor alpha ligand binding domain in complex with 4-hydroxytamoxifen (PDB ID: 3ERT) was used for the docking. A thorough analysis of the X-ray crystal structure of estrogen receptor revealed that the active site regions has flexible amino acid side chains and hence could accommodate different chemical scaffolds. The amino acid residues lining active site are: Phe404, Glu419, Leu428, Met343, Gly420, Met421, Leu525, Gly521, Thr347, Leu387, Asp351, Ala350, Glu353, Trp383, Arg394, Leu346, respectively. The protein was prepared using Molegro software. All bond orders and hybridization were assigned, hydrogen and other missing atoms were added to the residues and charges were assigned. The co-crystallized water molecules were excluded from docking. Cavities in the protein were evaluated by Cavity detection algorithm using Expanded Van der Waals molecular surface with default parameters such as minimum and maximum cavity volume set at 10 and 10000 Å, with 1.20 Å probe radius and grid resolution being 0.80 resulted in 5 cavities. A docking template was created using bound ligand, with a probe radius of 1.20 Å is used as template for docking external ligands within the active site space of protein. In this case, tamoxifen co-crystallized in ERa was set as ligand template and docking routine was performed using this template complexed in first cavity. 3ERT subjected to docking in triplicate in silico analysis using default parameters of Molegro resulted in RMSD less than 2 Å in all cases with average dock score -149.856 kcal/mol and RMSD 0.85 Å.

### Set-1: ERa ligands from literature

**Figure 1:**
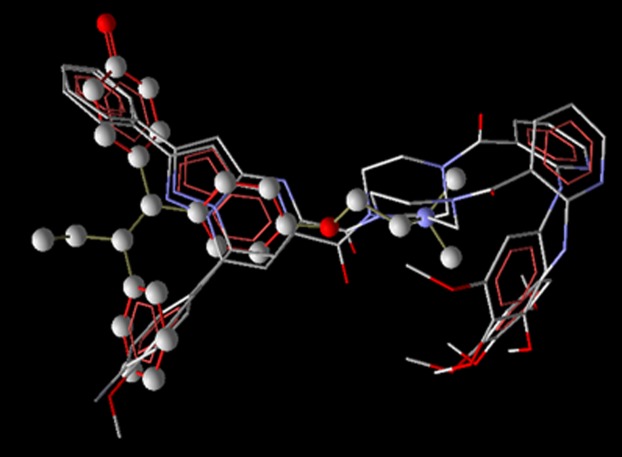
Structural superimposition of top 3 literature compounds Vstamoxifen (ball and stick model).

All 105 literature compounds (Table 1) converted into 3D formats are subjected to docking against ERa protein 3ERT using default parameters. Docking analysis resulted in varied dock scores, and compounds that exhibited better binding affinities than tamoxifen are given in Table 2. From Table 2, it is evidenced that nearly 31 compounds displayed better binding affinities than 3ERT bound tamoxifen (-149.856 kcal/mol). The maximum score obtained was - 175.282 kcal/mol for compound 11_6j. Interestingly, almost all compounds under 11 and 14 series displayed better affinities than tamoxifen. Compounds under 11 series represent pyrazolo[1,5- a]pyrimidine analogs whereas 3-aryl-4-anilino-2H-chromen-2-ones were reported under 14 series. The superimposed structures of top 3 compounds with tamoxifen are given in Figure 1 and the h-bond interactions are given in Table 3.

An electrostatic interaction was observed when the ligand interacted with oxygen atoms of Asp351. On the other hand, all other interacting amino acids displayed H-bond forces. Further, careful observations on the interacting amino acid residues revealed that pyrazolo[1,5-a]pyrimidine analogs under 11 series displayed major interactions with Thr347 whereas the 3-aryl-4- anilino-2H-chromen-2-ones reported under 14 series interacted majorly with His524 amino acid. The ERa bound tamoxifen displayed favourable interactions with Asp351 and Arg394, respectively. Similar interactions are observed with majority of the 14 series chromene derivatives.

### Set-2: ERa Non-tested ligands from literature

**Figure 2:**
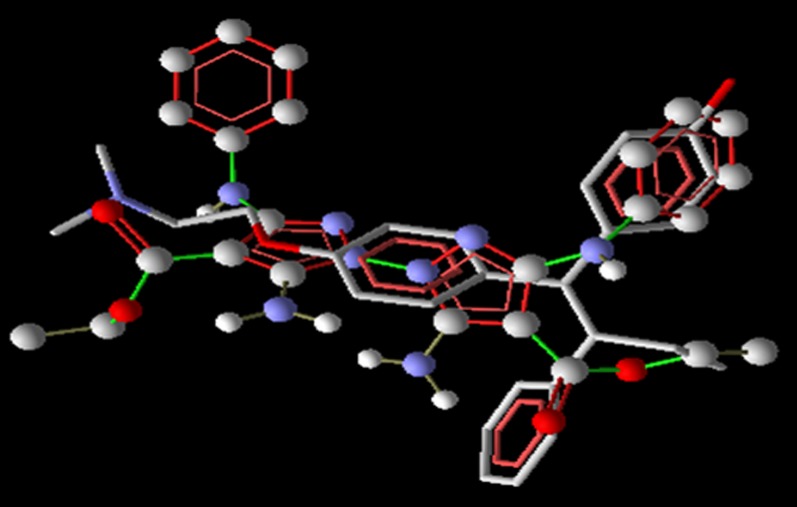
Overlap image of bipyrazole compound 2 (dock score - 175.937 kcal per mol) with ERa bound tamoxifen.

**Figure 3:**
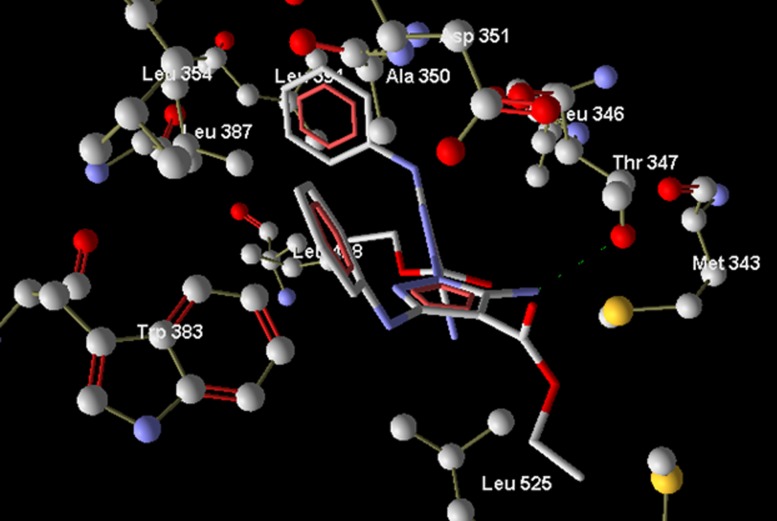
Bipyrazole compound 2 showing H-bond interaction with Thr347

**Figure 4:**
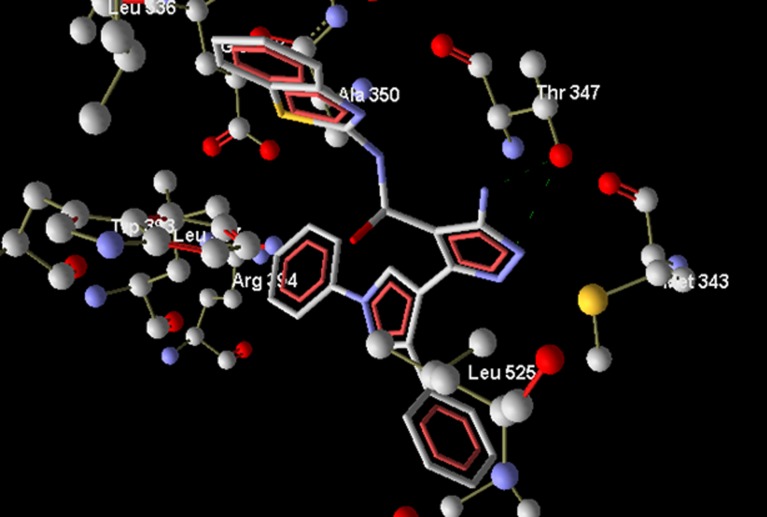
Bipyrazole compound 29 (dock score -167.1 kcal/mol) showing two H-bond interactions with Thr347

A thorough literature search was made on structural features of ligands that would fit into the active site region of ERa, which resulted in pyrazole, bipyrazole, thiazole, thiadiazoleetc scaffold analogs. Bipyrazoles are known to possess inhibitory properties against several classes of enzymes. Moreover, preliminary docking analysis revealed better inhibition of ERa with bipyrazoles. Other classes of compounds displayed reduced inhibition. Hence, bipyrazoles are considered for further analysis. Computational molecular docking and structural specificity of bipyrazoles as inhibitors of ERa Docking of all 34 bipyrazoles from literature was carried out to evaluate the best conformer based on the lowest docked energy (kcal/mol) (Table 4), in other words, it should possess highest affinity towards the binding site 42.From the bipyrazole Vs ERa docking analysis output, it is evidenced that the bipyrazoles are able to bind and fit into the geometrical space provided by the active site region of ERa. The binding orientations of all bipyrazoles were similar to the cocrystallized ligand, tamoxifen (Figure 2 ). The best compound 2 (ethyl 5-amino-1-(5-amino-3-anilino-4-ethoxycarbonyl-pyrazol-1-yl)-3- anilino-pyrazole-4-carboxylate) from Table-7 displayed a score of - 175.9 kcal per mol which is much better than the ERa bound ligand (- 149.8 kcal per mol). A favourable H-bond was formed with Thr347 (Figure 3 ) as observed with chromene derivatives. The next best compound 29 resulted in dock score (-167.1 kcal per mol), however two favourable H-bonds were found to interact with compound 29, via Thr347 (Figure 4 ).

### Set-3: Drugs from DrugBank Database

Owing to the output from bipyrazole dataset, which showed better inhibitory than tamoxifen, the next step utilized was to search DrugBank database because it was observed that certain drugs which are specific against a particular disease were found to be effective against other disease conditions as well, for example, Pioglitazone, a drug used for type 2 diabetes, may prevent recurrent stroke and heart attacks in people with insulin resistance but without diabetes 43-44. Several studies indicate that persons with type-2 diabetes are at higher risk of cancer of the pancreas, liver, endometrium, breast, colon, rectum and urinary bladder 45. however, the use of metformin was associated with decreased risk of the occurrence of various types of cancers, especially of pancreas and colon and hepatocellular carcinoma 46 evidence suggested that metformin might reduce breast cancer incidence in postmenopausal women 47 In another study, by screening already approved drugs, researchers identified calcium channel blockers, which are used to treat hypertension, can efficiently stop cancer cell invasion in vitro 48. Preliminary investigations revealed that Gleevec blocked the progression and development of rheumatoid arthritis in laboratory mice 49. Therefore, in this context DrugBank database was accessed to select 2035 FDA approved drugs and subjected to molecular docking. Analysis resulted in 15 drugs, which showed better binding affinities than ERa bound tamoxifen, tabulated in Table 5.

**Figure 5:**
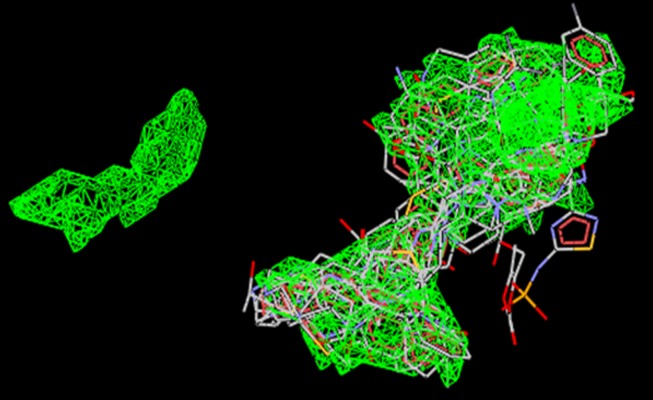
All 15 drugs superimposed within the active site of ERa

Table 5 represented better inhibitory values of various drugs intended for specific disease conditions when compared to ERa bound tamoxifen. The top best compound obtained from analysis was Cobicistat with binding energy, -187.123 kcal per mol. All drugs displayed H-bond interactions except Lomitapide and Ritonavir, which displayed Van der Waals interactions with ERa.Superimposition of all drugs within the active space of ERa is given in Figure 5 where it is evidenced that all drugs occupied clearly within the geometric space of the protein. From the table, out of 15 drugs, only 11 are finalized to consider for further analysis. This is because the four drugs viz., Bazedoxifene, Lapatinib, Raloxifene and Dabrafenib found to be anti-cancer drugs and hence omitted from the list.

### Consensus Scoring to enrich drugs active against ERa:

It has been reported recently that consensus scoring, which combines multiple scoring functions, leads to higher hit-rates in virtual library screening studies 50 and presented an idealized computer experiment to explore how consensus scoring works based on the assumption that the error of a scoring function is a random number in a normal distribution. Many studies suggested that implementing consensus-scoring approaches enhances the performance by compensating for the deficiencies of the scoring functions with each other 51, 52, 53 The possibility that several scoring methods might have their own strengths and weaknesses and combined use of more than one method might increase the overall signal-noise ratio and might perform better than the average of the individual scoring functions 54 presented computer-aided analysis where they implemented an intersection-based consensus approach to group few scoring functions. Stahl and Rarey 55 reported the performance of four scoring functions on seven target proteins.

Screening analysis of DrugBank database drugs against ERa resulted in 11 drugs and all these drugs are subjected to consensus scoring using 5 scoring schemes such as MolDock score of Molegro, mcule, SwissDock, Pose & Rank and DSX respectively. Here, we chose the 'rank-by-number' strategy to pool the output of multiple scoring functions. This is because, this strategy was reported to outperform the other techniques such as 'rank-by-rank' and 'rank by- vote' as the rank-by-number strategy summarized most of the information 56 Each scoring function was applied to generate three classes based on the obtained dock scores followed by ranking the best conformations. Classes were generated for all scoring functions and instead of taking an average, rank-bynumber technique 57 was employed to finalize best compounds. The ranks obtained from each of the scoring functions were added to give the rank-sum. The benefit of rank-by-number technique is that the each individual score involvement for a rank can certainly be split out for illustrative purposes 58. The rank sums obtained for 11 drugs against five scoring functions were in the range 5 to 15, with 5 being low rank and 15 being first and best rank, respectively (Table 6). Therefore, finally from 11 drugs, the top five compounds with rank-sums 15 - 12 (Lomitapide, Itraconazole, Cobicistat, Azilsartanmedoxomil, and Zafirlukast) are finalized. Further work shall be carried out to study their affinity of binding and inhibitory characteristics against ERa in a breast cancer cell line MCF-7.

## Conclusion

Molecular docking analysis carried out on a set of ERa inhibitors against 3ERT, complexed with 4-hydroxytamoxifen (-149.856 kcal/mol with RMSD 0.85 Å) resulted in better binding affinities than 3ERT bound tamoxifen for nearly 31 compounds with pyrazolo[1,5-a]pyrimidine and chromen-2-one derivatives. The best compound (-175.282 kcal/mol) was [2-(4-Fluoro-phenylamino)- pyridin-3-yl]-{4-[2-phenyl-7-(3,4,5-trimethoxy-phenyl)-pyrazolo[1,5- a]pyrimidine-5-carbonyl]-piperazin-1-yl}-methanone and favourable interactions were observed with Thr347. In our search to unearth entirely novel compounds, bipyrazole nucleus compounds were analyzed which resulted in with -175.9 kcal/mol binding affinity with the receptor and favourable H-bond interaction with Thr347. After realizing this novel inhibitor, 2035 FDA approved drugs from DrugBank database were screened to study their efficacy against ERa, resulted in 15 such drugs with binding affinities greater than tamoxifen ranging from -164.66 to -187.12 kcal per mol. After eliminating 4 anti-cancer drugs, the remaining 11 drugs are subjected to consensus scoring using MolDock score of Molegro, mcule, SwissDock, Pose & Rank and DSX. Consensus analysis resulted in top ranks for 5 drugs viz., Lomitapide, Itraconazole, Cobicistat, Azilsartanmedoxomil, and Zafirlukast, which were selected further to assess their experimental activity in an MCF-7 cell line.

## Conflict of Interest

Authors declare no conflict of interest.

## Figures and Tables

**Table 1:** Physico-chemical properties and related information of 105 literature compound data

T1	SMILES	MW	HBA	HBD	logP	RB
1_4d.mol	Oc1ccc(cc1)N(CC1CC1)c1ccc(cc1)O	255.34	2	2	3.6614	4
6_12.mol	O=C1CS[C@H](N1c1ccccc1)c1ccccc1	255.35	1	0	3.2436	2
estradiol.mol	O[C@@H]1CC[C@@H]2[C@@H]1CC[C@@H]1[C@@H]2CCc2cc(ccc21)O	258.39	2	2	3.5024	0
diethylstilbestrol.mol	CC/C(/c1ccc(cc1)O)=C(/CC)\c1ccc(cc1)O	268.38	2	2	4.794	5
1_4e.mol	CC(C)CCN(c1ccc(cc1)O)c1ccc(cc1)O	271.39	2	2	4.4894	5
1_4m.mol	Oc1ccc(cc1)N(c1ccccc1)c1ccc(cc1)O	277.34	2	2	4.6332	3
6_1.mol	Oc1ccc(cc1)[C@@H]1SCC(=O)N1c1ccc(cc1)O	287.35	3	2	2.6748	2
1_4j.mol	Oc1ccc(cc1)N(Cc1ccccc1)c1ccc(cc1)O	291.37	2	2	4.7281	4
5_2.mol	COc1cc2occ(c2cc1O)C(=O)/C=C/c1ccccc1	294.32	4	1	3.0895	5
1_4g.mol	Oc1ccc(cc1)N(CC1CCCCC1)c1ccc(cc1)O	297.43	2	2	4.8503	4
1_4l.mol	Oc1ccc(cc1)N(CC1CCCCC1)c1ccc(cc1)O	297.43	2	2	4.8503	4
6_4.mol	Oc1ccc(cc1)N1[C@@H](SCC1=O)c1ccc(c(c1)O)O	303.35	4	3	2.3904	2
6_5.mol	Oc1ccc(cc1)N1[C@@H](SCC1=O)c1cc(cc(c1)O)O	303.35	4	3	2.3904	2
6_6.mol	Oc1ccc(cc1)N1[C@@H](SCC1=O)c1ccc(cc1O)O	303.35	4	3	2.3904	2
6_11.mol	Cc1ccc(cc1)N1[C@@H](SCC1=O)c1ccc(cc1)Cl	303.82	1	0	4.2288	2
6_10.mol	Oc1ccc(cc1)N1[C@@H](SCC1=O)c1ccc(cc1)Cl	305.79	2	1	3.4772	2
1_4k.mol	Oc1ccc(cc1)CN(c1ccc(cc1)O)c1ccc(cc1)O	307.37	3	3	4.4437	4
1_4h.mol	Oc1ccc(cc1)N(CCC1CCCCC1)c1ccc(cc1)O	311.46	2	2	5.1743	5
5_5.mol	COc1ccc(cc1)\C=C\C(=O)c1coc2cc(c(cc21)O)F	312.31	4	1	3.229	5
3_vioxx.mol	CS(=O)(=O)c1ccc(cc1)C1=C(C(=O)OC1)c1ccccc1	314.37	4	0	2.2409	3
5_4.mol	Oc1cc2c(occ2C(=O)/C=C/c2cccc(c2)Cl)cc1F	316.72	3	1	3.9997	4
6_13.mol	Cc1ccc(cc1)N1[C@@H](SCC1=O)c1cccc2ccccc21	319.44	1	0	4.713	2
5_1.mol	COc1cc2occ(c2cc1O)C(=O)c1cccc(c1)NC(C)=O	325.34	5	2	1.5304	4
5_3.mol	COc1cc2occ(c2cc1O)C(=O)/C=C/c1cccc(c1)Cl	328.76	4	1	3.6075	5
8_11d.mol	Oc1ccc(cc1)C1=C(c2ccccc2)C2(OC1=O)C=CC(=O)C=C2	330.35	4	1	2.793	2
6_7.mol	COc1ccc(c(c1)OC)[C@@H]1SCC(=O)N1c1ccc(cc1)O	331.41	4	1	2.4538	4
6_9.mol	COc1ccc(cc1OC)[C@@H]1SCC(=O)N1c1ccc(cc1)O	331.41	4	1	2.4538	4
8_11b.mol	Fc1ccc(cc1)C1=C(c2ccccc2)C2(OC1=O)C=CC(=O)C=C2	332.34	3	0	3.2169	2
4_4m.mol	CC(C)(C)\C=C\c1c(onc1c1ccc(cc1)O)c1ccc(cc1)O	335.43	4	2	5.6743	5
5_7.mol	COc1cc2occ(c2cc1O)C(=O)/C=C/c1ccc2c(c1)OCO2	338.33	6	1	2.424	5
8_11l.mol	O=C1C=CC2(OC(=O)C(=C2c2ccccc2)c2ccc(cc2)C#N)C=C1	339.36	4	0	2.9424	2
8_11f.mol	COc1ccc(cc1)C1=C(c2ccccc2)C2(OC1=O)C=CC(=O)C=C2	344.38	4	0	2.8247	3
8_11g.mol	OCc1ccc(cc1)C1=C(c2ccccc2)C2(OC1=O)C=CC(=O)C=C2	344.38	4	1	2.5421	3
8_11n.mol	O=Cc1sc(cc1)C1=C(c2ccccc2)C2(OC1=O)C=CC(=O)C=C2	348.38	4	0	1.8088	3
3_9b.mol	CC(c1ccc(cc1)S(C)(N)=O)=C(c1ccccc1)c1ccccc1	348.51	2	1		5
8_11c.mol	O=C1OC2(C=CC(=O)C=C2)C(=C1c1oc2ccccc2c1)c1ccccc1	354.37	4	0	2.7922	2
4_4a.mol	Oc1ccc(cc1)c1onc(c1/C=C/c1ccccc1)c1ccc(cc1)O	355.41	4	2	5.7315	5
8_11k.mol	O=C1C=CC2(OC(=O)C(=C2c2ccccc2)c2ccc3c(c2)OCO3)C=C1	358.36	5	0	2.4119	2
8_11m.mol	OC(=O)c1ccc(cc1)C1=C(c2ccccc2)C2(OC1=O)C=CC(=O)C=C2	358.36	5	1	2.7758	3
8_11e.mol	[O-][N+](=O)c1ccc(cc1)C1=C(c2ccccc2)C2(OC1=O)C=CC(=O)C=C2	359.35	5	0	3.031	2
6_14.mol	COc1cc(cc(c1OC)OC)[C@@H]1SCC(=O)N1c1ccc(cc1)C	359.47	4	0	2.9527	5
6_8.mol	COc1cc(cc(c1OC)OC)[C@@H]1SCC(=O)N1c1ccc(cc1)O	361.44	5	1	2.2011	5
8_11i.mol	COc1ccc(cc1C1=C(c2ccccc2)C2(OC1=O)C=CC(=O)C=C2)F	362.37	4	0	2.9642	3
4_4c.mol	Cc1ccc(cc1)\C=C\c1c(onc1c1ccc(cc1)O)c1ccc(cc1)O	369.44	4	2	6.1987	5
1_3.mol	Oc1ccc(cc1)C(c1ccc(cc1)O)=C(CC(F)(F)F)c1ccccc1	370.39	2	2	5.8763	5
5_6.mol	COc1cc2occ(c2cc1O)C(=O)/C=C/c1ccc(cc1)c1ccccc1	370.42	4	1	4.7739	6
8_11a.mol	O=C1OC2(C=CC(=O)C=C2)C(=C1c1sc2ccccc2c1)c1ccccc1	370.43	3	0	3.1355	2
4_4h.mol	Oc1ccc(cc1)\C=C\c1c(onc1c1ccc(cc1)O)c1ccc(cc1)O	371.41	5	3	5.4471	5
4_4i.mol	Oc1ccc(cc1)c1onc(c1/C=C/c1cccc(c1)O)c1ccc(cc1)O	371.41	5	3	5.4471	5
4_4d.mol	Oc1ccc(cc1)c1onc(c1/C=C/c1ccc(cc1)F)c1ccc(cc1)O	373.4	4	2	5.871	5
4_4j.mol	CCCCCCC\C=C\c1c(onc1c1ccc(cc1)O)c1ccc(cc1)O	377.52	4	2	6.8921	10
hydroxytamoxifen.mol	CC\C(\c1ccccc1)=C(/c1ccc(cc1)O)\c1ccc(cc1)OCCN(C)C	387.56	3	1	5.6257	9
4_4e.mol	Oc1ccc(cc1)c1onc(c1/C=C/c1ccc(cc1)Cl)c1ccc(cc1)O	389.85	4	2	6.2495	5
3_2.mol	CCCCC(c1ccc(cc1)S(C)(=O)=O)=C(c1ccccc1)c1ccccc1	390.57	2	0	6.1213	8
3_9a.mol	CCCCC(c1ccc(cc1)S(C)(N)=O)=C(c1ccccc1)c1ccccc1	390.6	2	1		8
4_4k.mol	CCCCCCCC\C=C\c1c(onc1c1ccc(cc1)O)c1ccc(cc1)O	391.55	4	2	7.2884	11
8_11h.mol	COc1cc(cc(c1OC)OC)C1=C(c2ccccc2)C2(OC1=O)C=CC(=O)C=C2	404.44	6	0	2.3193	5
4_4l.mol	CCCCCCCCC\C=C\c1c(onc1c1ccc(cc1)O)c1ccc(cc1)O	405.58	4	2	7.6847	12
3_8a.mol	CCCCC(c1ccc(cc1)S(C)(=O)NC#N)=C(c1ccccc1)c1ccccc1	415.61	3	1		9
4_4f.mol	Oc1ccc(cc1)c1onc(c1/C=C/c1ccc(cc1)C(F)(F)F)c1ccc(cc1)O	423.41	4	2	6.6143	5
4_4g.mol	Oc1ccc(cc1)c1onc(c1/C=C/c1cccc(c1)C(F)(F)F)c1ccc(cc1)O	423.41	4	2	6.6143	5
1_4i.mol	Oc1ccc(cc1)N(C[C@@]12C[C@@H]3C[C@@H](C[C@@](Br)(C3)C1)C2)c1ccc(cc1)O	428.4	2	2	5.3113	4
14_15a.mol	COc1ccc(cc1)C1=C(Nc2ccc(cc2)OCCN(C)C)c2ccccc2OC1=O	430.54	5	1	3.1602	8
8_11j.mol	COc1c(cc(cc1C1=C(c2ccccc2)C2(OC1=O)C=CC(=O)C=C2)C)Br	437.3	4	0	4.0837	3
14_18a.mol	COc1ccc(cc1)C1=C(Nc2ccc(cc2)OCCN(C)C)c2ccc(cc2OC1=O)O	446.54	6	2	2.8758	8
14_15c.mol	COc1ccc(cc1)C1=C(Nc2ccc(cc2)OCCN2CCCC2)c2ccccc2OC1=O	456.58	5	1	3.4858	8
14_15b.mol	CCN(CC)CCOc1ccc(cc1)NC1=C(C(=O)Oc2ccccc21)c1ccc(cc1)OC	458.6	5	1	3.8452	10
14_16a.mol	COc1ccc(cc1)C1=C(Nc2ccc(cc2)OCCN(C)C)c2ccc(cc2OC1=O)OC	460.57	6	1	2.9075	9
14_15d.mol	COc1ccc(cc1)C1=C(Nc2ccc(cc2)OCCN2CCCCC2)c2ccccc2OC1=O	470.61	5	1	3.8821	8
14_15e.mol	COc1ccc(cc1)C1=C(Nc2ccc(cc2)OCCN2CCOCC2)c2ccccc2OC1=O	472.58	6	1	2.8176	8
14_18c.mol	COc1ccc(cc1)C1=C(Nc2ccc(cc2)OCCN2CCCC2)c2ccc(cc2OC1=O)O	472.58	6	2	3.2014	8
14_18b.mol	CCN(CC)CCOc1ccc(cc1)NC1=C(C(=O)Oc2cc(ccc21)O)c1ccc(cc1)OC	474.6	6	2	3.5608	10
14_15f.mol	COc1ccc(cc1)C1=C(Nc2ccc(cc2)OCCN2CCN(C)CC2)c2ccccc2OC1=O	485.63	6	1	2.9615	8
14_16c.mol	COc1ccc(cc1)C1=C(Nc2ccc(cc2)OCCN2CCCC2)c2ccc(cc2OC1=O)OC	486.61	6	1	3.2331	9
14_18d.mol	COc1ccc(cc1)C1=C(Nc2ccc(cc2)OCCN2CCCCC2)c2ccc(cc2OC1=O)O	486.61	6	2	3.5977	8
14_18e.mol	COc1ccc(cc1)C1=C(Nc2ccc(cc2)OCCN2CCOCC2)c2ccc(cc2OC1=O)O	488.58	7	2	2.5332	8
14_16b.mol	CCN(CC)CCOc1ccc(cc1)NC1=C(C(=O)Oc2cc(ccc21)OC)c1ccc(cc1)OC	488.63	6	1	3.5925	11
14_16d.mol	COc1ccc(cc1)C1=C(Nc2ccc(cc2)OCCN2CCCCC2)c2ccc(cc2OC1=O)OC	500.64	6	1	3.6294	9
14_18f.mol	COc1ccc(cc1)C1=C(Nc2ccc(cc2)OCCN2CCN(C)CC2)c2ccc(cc2OC1=O)O	501.63	7	2	2.6771	8
14_16e.mol	COc1ccc(cc1)C1=C(Nc2ccc(cc2)OCCN2CCOCC2)c2ccc(cc2OC1=O)OC	502.61	7	1	2.5649	9
14_16f.mol	COc1ccc(cc1)C1=C(Nc2ccc(cc2)OCCN2CCN(C)CC2)c2ccc(cc2OC1=O)OC	515.66	7	1	2.7088	9
11_6a.mol	Fc1ccc(cc1)C1=CC(=Nc2cc(nn21)c1ccccc1)C(=O)N1CCN(CC1)C(=O)c1cccnc1Nc1ccccc1	597.7	5	1	4.5686	6
11_6c.mol	COc1ccc(cc1)C1=CC(=Nc2cc(nn21)c1ccccc1)C(=O)N1CCN(CC1)C(=O)c1cccnc1Nc1ccccc1	609.74	6	1	4.1764	7
11_6f.mol	Fc1ccc(cc1)Nc1ncccc1C(=O)N1CCN(CC1)C(=O)C1=Nc2cc(nn2C(=C1)c1ccc(cc1)F)c1ccccc1	615.69	5	1	4.7081	6
11_6h.mol	COc1ccc(cc1)C1=CC(=Nc2cc(nn21)c1ccccc1)C(=O)N1CCN(CC1)C(=O)c1cccnc1Nc1ccc(cc1)F	627.73	6	1	4.3159	7
11_6k.mol	COc1ccc(cc1)Nc1ncccc1C(=O)N1CCN(CC1)C(=O)C1=Nc2cc(nn2C(=C1)c1ccc(cc1)F)c1ccccc1	627.73	6	1	4.3159	7
11_6d.mol	COc1ccc(cc1OC)C1=CC(=Nc2cc(nn21)c1ccccc1)C(=O)N1CCN(CC1)C(=O)c1cccnc1Nc1ccccc1	639.771	7	1	3.9237	8
11_6m.mol	COc1ccc(cc1)Nc1ncccc1C(=O)N1CCN(CC1)C(=O)C1=Nc2cc(nn2C(=C1)c1ccc(cc1)OC)c1ccccc1	639.771	7	1	3.9237	8
11_6b.mol	Clc1ccc(cc1Cl)C1=CC(=Nc2cc(nn21)c1ccccc1)C(=O)N1CCN(CC1)C(=O)c1cccnc1Nc1ccccc1	648.59	5	1	5.4651	6
11_6i.mol	COc1ccc(cc1OC)C1=CC(=Nc2cc(nn21)c1ccccc1)C(=O)N1CCN(CC1)C(=O)c1cccnc1Nc1ccc(cc1)F	657.76	7	1	4.0632	8
11_6p.mol	COc1ccc(cc1OC)Nc1ncccc1C(=O)N1CCN(CC1)C(=O)C1=Nc2cc(nn2C(=C1)c1ccc(cc1)F)c1ccccc1	657.76	7	1	4.0632	8
11_6g.mol	Fc1ccc(cc1)Nc1ncccc1C(=O)N1CCN(CC1)C(=O)C1=Nc2cc(nn2C(=C1)c1ccc(c(c1)Cl)Cl)c1ccccc1	666.58	5	1	5.6046	6
11_6e.mol	COc1cc(cc(c1OC)OC)C1=CC(=Nc2cc(nn21)c1ccccc1)C(=O)N1CCN(CC1)C(=O)c1cccnc1Nc1ccccc1	669.801	8	1	3.671	9
11_6n.mol	COc1ccc(cc1)Nc1ncccc1C(=O)N1CCN(CC1)C(=O)C1=Nc2cc(nn2C(=C1)c1ccc(c(c1)OC)OC)c1ccccc1	669.801	8	1	3.671	9
11_6r.mol	COc1ccc(cc1)C1=CC(=Nc2cc(nn21)c1ccccc1)C(=O)N1CCN(CC1)C(=O)c1cccnc1Nc1ccc(c(c1)OC)OC	669.801	8	1	3.671	9
11_6l.mol	COc1ccc(cc1)Nc1ncccc1C(=O)N1CCN(CC1)C(=O)C1=Nc2cc(nn2C(=C1)c1ccc(c(c1)Cl)Cl)c1ccccc1	678.62	6	1	5.2124	7
11_6j.mol	COc1cc(cc(c1OC)OC)C1=CC(=Nc2cc(nn21)c1ccccc1)C(=O)N1CCN(CC1)C(=O)c1cccnc1Nc1ccc(cc1)F	687.791	8	1	3.8105	9
11_6u.mol	COc1cc(cc(c1OC)OC)Nc1ncccc1C(=O)N1CCN(CC1)C(=O)C1=Nc2cc(nn2C(=C1)c1ccc(cc1)F)c1ccccc1	687.791	8	1	3.8105	9
11_6o.mol	COc1ccc(cc1)Nc1ncccc1C(=O)N1CCN(CC1)C(=O)C1=Nc2cc(nn2C(=C1)c1cc(c(c(c1)OC)OC)OC)c1ccccc1	699.831	9	1	3.4183	10
11_6s.mol	COc1ccc(cc1OC)Nc1ncccc1C(=O)N1CCN(CC1)C(=O)C1=Nc2cc(nn2C(=C1)c1ccc(c(c1)OC)OC)c1ccccc1	699.831	9	1	3.4183	10
11_6w.mol	COc1ccc(cc1)C1=CC(=Nc2cc(nn21)c1ccccc1)C(=O)N1CCN(CC1)C(=O)c1cccnc1Nc1cc(c(c(c1)OC)OC)OC	699.831	9	1	3.4183	10
11_6q.mol	COc1ccc(cc1OC)Nc1ncccc1C(=O)N1CCN(CC1)C(=O)C1=Nc2cc(nn2C(=C1)c1ccc(c(c1)Cl)Cl)c1ccccc1	708.651	7	1	4.9597	8
11_6t.mol	COc1ccc(cc1OC)Nc1ncccc1C(=O)N1CCN(CC1)C(=O)C1=Nc2cc(nn2C(=C1)c1cc(c(c(c1)OC)OC)OC)c1ccccc1	729.861	10	1	3.1656	11
11_6x.mol	COc1ccc(cc1OC)C1=CC(=Nc2cc(nn21)c1ccccc1)C(=O)N1CCN(CC1)C(=O)c1cccnc1Nc1cc(c(c(c1)OC)OC)OC	729.861	10	1	3.1656	11
11_6v.mol	COc1cc(cc(c1OC)OC)Nc1ncccc1C(=O)N1CCN(CC1)C(=O)C1=Nc2cc(nn2C(=C1)c1ccc(c(c1)Cl)Cl)c1ccccc1	738.681	8	1	4.7079

**Table 2:** Compounds which exhibited better binding affinities than bound tamoxifen

S. No	Ligand	MolDock Score
1	11_6j.mol	-175.282
2	11_6o.mol	-172.882
3	14_16c.mol	-171.234
4	11_6h.mol	-169.719
5	14_15e.mol	-168.139
6	11_6e.mol	-167.14
7	14_16d.mol	-165.673
8	11_6g.mol	-165.019
9	14_15c.mol	-164.805
10	11_6k.mol	-164.018
11	14_18e.mol	-162.91
12	11_6d.mol	-162.147
13	11_6t.mol	-161.625
14	14_18f.mol	-160.374
15	14_18d.mol	-159.463
16	11_6n.mol	-158.725
17	11_6i.mol	-158.478
18	14_18a.mol	-157.811
19	11_6a.mol	-156.748
20	11_6c.mol	-156.4
21	11_6s.mol	-156.129
22	11_6q.mol	-156.068
23	11_6p.mol	-155.936
24	11_6f.mol	-155.614
25	11_6b.mol	-154.125
26	14_15a.mol	-154.078
27	11_6x.mol	-153.768
28	11_6l.mol	-153.718
29	14_18b.mol	-153.633
30	11_6m.mol	-153.504
31	14_16a.mol	-152.413
32	Tamoxifen	-149.856

**Table 3:** H-bond interactions of top 31 compounds

S. No	Ligand	MolDock Score	H-bond interacting amino acid residues	H-bond energy (kcal/mol)
1	11_6j.mol	-175.282	Cys530, Thr347	-4.617
2	11_6o.mol	-172.882	Cys530, Thr347	-5
3	14_16c.mol	-171.234	Arg394, Glu353, His524	-5.644
4	11_6h.mol	-169.719	Thr347	-0.2
5	14_15e.mol	-168.139	Arg394, Glu353, His524	-5.336
6	11_6e.mol	-167.14	Thr347, Asp351	-3.02
7	14_16d.mol	-165.673	Arg394, His524	-3.141
8	11_6g.mol	-165.019	Thr347	-2.455
9	14_15c.mol	-164.805	His524	-1.214
10	11_6k.mol	-164.018	Thr347	-2.059
11	14_18e.mol	-162.91	Arg394, Glu353, His524	-5.729
12	11_6d.mol	-162.147	Cys530, Thr347	-2.789
13	11_6t.mol	-161.625	Leu536, Thr347, His524	-4.251
14	14_18f.mol	-160.374	Arg394, Glu353, His524	-5.331
15	14_18d.mol	-159.463	Arg394, His524	-3.6
16	11_6n.mol	-158.725	Thr347	-2.333
17	11_6i.mol	-158.478	Thr347	-2.388
18	14_18a.mol	-157.811	Arg394, Glu353, His524, Leu387, Asp351	-6.595
19	11_6a.mol	-156.748	Thr347	-2.5
20	11_6c.mol	-156.4	Thr347	-2.369
21	11_6s.mol	-156.129	Thr347, Cys530	-3.623
22	11_6q.mol	-156.068	Thr347	-2.227
23	11_6p.mol	-155.936	Thr347	-1.414
24	11_6f.mol	-155.614	Thr347	-2.5
25	11_6b.mol	-154.125	Thr347	-1.064
26	14_15a.mol	-154.078	Asp351, His524	-1.176
27	11_6x.mol	-153.768	Arg394	-1.237
28	11_6l.mol	-153.718	Thr347, Cys530	-2.682
29	14_18b.mol	-153.633	Glu353, Arg394, His524	-5.869
30	11_6m.mol	-153.504	Thr347	-2.073
31	14_16a.mol	-152.413	Asp351, Arg394, His524	-3.214
32	Tamoxifen	-149.856	Asp351, Arg394	-2.5

**Table 4:** IUPAC names, SMILES notation and molecular dock scores in kcal/mol of 34 bipyrazole class of compounds

ID	IUPAC Name	SMILES	Dock Score (kcal/mol)
1	ethyl 5-amino-3-anilino-1H-pyrazole-4-carboxylate	CCOC(=O)c1c(N)[nH]nc1Nc2ccccc2	-105.689
2	ethyl 5-amino-1-(5-amino-3-anilino-4-ethoxycarbonyl-pyrazol-1-yl)-3-anilino-pyrazole-4-carboxylate	CCOC(=O)c1c(N)n(nc1Nc2ccccc2)n3nc (Nc4ccccc4)c(C(=O)OCC)c3N	-175.937
3	ethyl 5-amino-1-(4-chloro-4-ethoxycarbonyl-5-oxo-1H-pyrazol-3-yl)-3-ethoxy-pyrazole-4-carboxylate	CCOC(=O)c1c(N)n(nc1OCC)C2=NNC(=O) C2(Cl)C(=O)OCC	-137.361
4	5-(4-chlorophenyl)-4-(4-cyanopyrazol-1-yl)-N-(4-phenylphenyl)-3,4-dihydropyrazole-2-carboxamide	Clc1ccc(cc1)C2=NN(CC2n3cc(cn3)C#N)C(=O) Nc4ccc(cc4)c5ccccc5	-139.765
5	1-(1,5-diphenylpyrazol-4-yl)-3,5-dimethyl-pyrazole	Cc1cc(C)n(n1)c2cnn(c3ccccc3)c2c4ccccc4	-131.507
6	methyl 4-(3,5-dimethylpyrazol-1-yl)-5-phenyl-pyrazole-1-carboxylate	COC(=O)n1ncc(c1c2ccccc2)n3nc(C)cc3C	-120.717
7	1-tert-butyl-4-(3,5-dimethylpyrazol-1-yl)-5-phenyl-pyrazole	Cc1cc(C)n(n1)c2cnn(c2c3ccccc3)C(C)(C)C	-117.359
8	bis(2-adamantyl)-[2-[1-(4-methoxyphenyl)-3,5-diphenyl-pyrazol-4-yl]pyrazol-3-yl]phosphane	COc1ccc(cc1)n2nc(c3ccccc3)c(c2c4ccccc4)n5nccc5P(C6C7CC8CC(CC6C8)C7) C9C%10CC%11CC(CC9C%11) C%10	-146.054
9	dicyclohexyl-[2-[1-(4-methoxyphenyl)-3,5-diphenyl-pyrazol-4-yl]pyrazol-3-yl]phosphane	COc1ccc(cc1)n2nc(c3ccccc3)c(c2c4ccccc4)n5nccc5P (C6CCCCC6)C7CCCCC7	-148.556
10	ditert-butyl-[2-[1-(4-methoxyphenyl)-3,5-diphenyl-pyrazol-4-yl]pyrazol-3-yl]phosphane	COc1ccc(cc1)n2nc(c3ccccc3)c(c2c4ccccc4)n5nccc5P(C(C)(C)C)C(C)(C)C	-147.159
11	4-chloro-1-(3,5-dinitro-1H-pyrazol-4-yl)-5-nitro-pyrazole	[O-][N+](=O)c1n[nH]c(c1n2ncc(Cl)c2[N+](=O)[O-])[N+](=O)[O-]	-110.157
12	1-(3,5-dinitro-1H-pyrazol-4-yl)-4,5-dinitro-pyrazole	[O-][N+](=O)c1cnn(c1[N+](=O)[O-])c2c(n[nH]c2[N+](=O)[O-])[N+](=O)[O-]	-117.658
13	1-methyl-3,4-dinitro-5-(3-nitropyrazol-1-yl)pyrazole	Cn1nc(c(c1n2ccc(n2)[N+](=O)[O-])[N+](=O)[O-])[N+](=O)[O-]	-109.876
14	1-methyl-3,4-dinitro-5-(4-nitropyrazol-1-yl)pyrazole	Cn1nc(c(c1n2cc(cn2)[N+](=O)[O-])[N+](=O)[O-])[N+](=O)[O-]	-107.758
15	N-[1-(4-methoxyphenyl)-3-methyl-5-pyrazol-1-yl-pyrazol-4-yl]methanesulfonamide	COc1ccc(cc1)n2nc(C)c(NS(=O)(=O)C)c2n3cccn3	-125.453
16	N-[1-(4-bromophenyl)-3-methyl-5-pyrazol-1-yl-pyrazol-4-yl]methanesulfonamide	Cc1nn(c2ccc(Br)cc2)c(c1NS(=O)(=O)C)n3cccn3	-120.706
17	N-[1-(4-chlorophenyl)-3-methyl-5-pyrazol-1-yl-pyrazol-4-yl]methanesulfonamide	Cc1nn(c2ccc(Cl)cc2)c(c1NS(=O)(=O)C)n3cccn3	-118.696
18	N-[1-(4-fluorophenyl)-3-methyl-5-pyrazol-1-yl-pyrazol-4-yl]methanesulfonamide	Cc1nn(c2ccc(F)cc2)c(c1NS(=O)(=O)C)n3cccn3	-123.955
19	N-[3-methyl-1-(4-nitrophenyl)-5-pyrazol-1-yl-pyrazol-4-yl]methanesulfonamide	Cc1nn(c2ccc(cc2)[N+](=O)[O-])c(c1NS(=O)(=O)C) n3cccn3	-121.433
20	ethyl 5-amino-1-(5-methyl-4-nitro-2-phenyl-pyrazol-3-yl)pyrazole-4-carboxylate	CCOC(=O)c1cnn(c1N)c2c(c(C)nn2c3ccccc3)[N+] (=O)[O-]	-133.582
21	3-acetyl-1-(4-bromo-3-phenyl-1H-pyrazol-5-yl)-5-phenyl-pyrazole-4-carbonitrile	CC(=O)c1nn(c(c2ccccc2)c1C#N)c3[nH]nc(c3Br) c4ccccc4	-148.595
22	ethyl 3-acetyl-5-amino-1-(4-bromo-3-phenyl-1H-pyrazol-5-yl)pyrazole-4-carboxylate	CCOC(=O)c1c(N)n(nc1C(=O)C)c2[nH]nc(c2Br) c3ccccc3	-113.874
23	1-(4-nitrophenyl)-3-[1-(4-nitrophenyl)-5-propyl-pyrazol-3-yl]-5-propyl-pyrazole	CCCc1cc(nn1c2ccc(cc2)[N+](=O)[O-])c3cc(CCC)n (n3)c4ccc(cc4)[N+](=O)[O-]	-154.386
24	5-isopropyl-3-[5-isopropyl-1-(4-nitrophenyl)pyrazol-3-yl]-1-(4-nitrophenyl)pyrazole	CC(C)c1cc(nn1c2ccc(cc2)[N+](=O)[O-])c3cc(C(C)C)n(n3)c4ccc(cc4)[N+](=O)[O-]	-154.361
25	5-[5-carbamoyl-1-(2,4-dichlorophenyl)-4H-pyrazol-3-yl]-2-(2,4-dichlorophenyl)pyrazole-3-carboxamide	NC(=O)C1=[N](N=C(C1)c2cc(C(=O)N)n(n2)c3ccc(Cl)cc3Cl) c4ccc(Cl)cc4Cl	-130.783
26	2-[5-[5-(1,3-benzothiazol-2-yl)-1,4-bis(4-chlorophenyl)pyrazol-3-yl]-2,4-bis(4-chlorophenyl)-4H-pyrazol-3-yl]-1,3-benzothiazole	Clc1ccc(cc1)C2C(=N[N](=C2c3nc4ccccc4s3)c5ccc(Cl)cc5)c6nn(c7ccc(Cl)cc7)c (c8nc9cccc c9s8) c6c%10ccc(Cl)cc%10	-138.603
27	[2-(4-chlorophenyl)-5-[1-(4-chlorophenyl)-5-(2-hydroxybenzoyl)-4-phenyl-4H-pyrazol-3-yl]-4-phenyl-pyrazol-3-yl]-(2-hydroxyphenyl)methanone	Oc1ccccc1C(=O)C2=[N](N=C(C2c3ccccc3)c4nn(c5ccc(Cl)cc5)c(C(=O)c6ccccc6O)c4c7ccccc7)c8ccc(Cl)cc8	-140.477
28	1-(4-chlorophenyl)-5-phenyl-3-(1H-pyrazol-3-yl)pyrazole-4-carbohydrazide	NNC(=O)c1c(nn(c2ccc(Cl)cc2)c1c3ccccc3)c4cc[nH]n4	-137.395
29	4-[(4Z)-5-amino-4-[(4-bromophenyl)methylene]pyrazol-3-yl]-1,5-dimethyl-2-phenyl-pyrazol-3-one	CN1N(C(=O)C(=C1C)C2=NN=C(N)/C/2=C\c3ccc(Br)cc3)c4ccccc4	-167.179
30	(E)-3-(2-hydroxyphenyl)-1-[1-phenyl-3-(2-thienyl)pyrazol-4-yl]prop-2-en-1-one	Oc1ccccc1\C=C\C(=O)c2cn(nc2c3cccs3)c4ccccc4	-98.6882
31	5-methyl-4-[5-(4-oxochromen-3-yl)-4,5-dihydro-1H-pyrazol-3-yl]-1,2-dihydropyrazol-3-one	CC1=C(C(=O)NN1)C2=NNC(C2)C3=COc4ccccc4C3=O	-138.46
32	5-amino-N-(1,3-benzothiazol-2-yl)-3-(1,3-diphenylpyrazol-4-yl)-1H-pyrazole-4-carboxamide	Nc1[nH]nc(c2cn(nc2c3ccccc3)c4ccccc4)c1C(=O)Nc5nc6ccccc6s5	-145.914
33	3-(5-hydroxy-3-methyl-1-phenyl-pyrazol-4-yl)-1H-pyrazole-5-carbohydrazide	Cc1nn(c(O)c1c2cc([nH]n2)C(=O)NN)c3ccccc3	-117.006
34	diethyl 2-(4-bromophenyl)-5-(4-cyano-5-methyl-2-phenyl-pyrazol-3-yl)pyrazole-3,4-dicarboxylate	CCOC(=O)c1c(nn(c2ccc(Br)cc2)c1C(=O)OCC)c3c(C#N)c(C)nn3c4ccccc4	-121.309

**Table 5:** Screening result of DrugBank database against ERa showing binding affinities (kcal/mol).

DrugBank ID	Binding affinity (kcal/mol)	Drug Name	Interaction Type	Interacting Residues	Drug Indication, disease and related information
DB09065	-187.123	Cobicistat	H-bonding	Arg394, Cys530	Cobicistat is a CYP3A inhibitor
DB08827	-185.233	Lomitapide	Van der Waals	No interactions	Used in homozygous familial hypercholesterolemia (HoFH) patients
DB01167	-180.646	Itraconazole	H-bonding	Thr347, Cys530	For the treatment of the fungal infections
DB06809	-178.689	Plerixafor	H-bonding	Glu353	Used in combination with granulocyte-colony stimulating factor (G-CSF, filgrastim) in patients with non-Hodgkin�s lymphoma (NHL) and multiple myeloma (MM).
DB08822	-173.473	Azilsartanmedoxomil	H-bonding	Thr347, His524	Treatment of hypertension (alone or as an adjunct).
DB00549	-172.426	Zafirlukast	H-bonding	Thr347	For the prophylaxis and chronic treatment of asthma.
DB06401	-170.261	Bazedoxifene	H-bonding	Gly420, His524, Leu387, Arg394	Bazedoxifene is a third generation selectiveestrogen receptor modulator (SERM).
DB01259	-169.171	Lapatinib�	H-bonding	Thr347, Asp351	Indicated in combination with capecitabine for the treatment of patients with advanced or metastatic breast cancer
DB00430	-166.876	Cefpiramide	H-bonding	Thr347, Asp351, Leu525	For treatment of severe infections caused by susceptible bacteria such as P. aeruginosa.
DB01264	-165.672	Darunavir	H-bonding	Leu346, Thr347	Darunavir, co-administered with ritonavir is indicated for the treatment of HIV infection
DB00503	-165.18	Ritonavir	Van der Waals	No interactions	Indicated in combination with other antiretroviral agents for the treatment of HIV-1 infection.
DB01263	-164.662	Posaconazole	H-bonding	Glu353, Leu387, Cys530	For prophylaxis of invasive Aspergillus and Candida infections
DB00481	-163.664	Raloxifene	H-bonding	Arg394, Glu353, Gly521, Gly420, His524	A second generation selective estrogen receptor modulator (SERM), for the prevention and treatment of osteoporosis in post-menopausal women
DB08912	-163.634	Dabrafenib	H-bonding	Gly521	Indicated for the treatment of patients with unresectable or metastatic melanoma.
DB06590	-163.214	Ceftarolinefosamil	H-bonding	Met343, Thr347, Cys530	Ceftarolinefosamil is a cephalosporin antibacterial.

**Table 6:** DrugBank drugs and corresponding scores of five scoring functions with rank-sum technique

Drug Name	DrugBank ID	DSX online	Rank	Pose & Rank	Rank	MolDock	Rank	mcule	Rank	Swiss	Rank	Rank-Sum
Cobicistat	DB09065	-124	2	-52.01	3	-187.123	3	-8	1	-10.08	3	12
Lomitapide	DB08827	-166	3	-49.06	3	-185.233	3	-10.3	3	-9.77	3	15
Itraconazole	DB01167	-125	2	-44.55	3	-180.646	3	-10.4	3	-9.52	3	14
Plerixafor	DB06809	-105	1	-25.77	1	-178.689	2	-9.6	3	-9.87	3	10
Azilsartanmedoxomil	DB08822	-107	2	-42.56	2	-173.473	2	-9.7	3	-9.06	3	12
Zafirlukast	DB00549	-137	3	-46.05	3	-172.426	2	-9.3	2	-8.36	2	12
Cefpiramide	DB00430	-96	1	-31.8	1	-166.876	1	-8.2	1	-6.83	1	5
Darunavir	DB01264	-119	2	-42.25	2	-165.672	1	-8.1	1	-7.82	1	7
Ritonavir	DB00503	-74	1	-26.46	1	-165.18	1	-7.6	1	-7.17	1	5
Posaconazole	DB01263	-116	2	-26.43	1	-164.662	1	-7.9	1	-7.4	1	6
Ceftarolinefosamil	DB06590	-102	1	-25.22	1	-163.214	1	-7.2	1	-7.72	1	5
